# Persistent Cigarette Smoking Attenuates Plaque Stabilization in Response to Lipid-Lowering Therapy: A Serial Optical Coherence Tomography Study

**DOI:** 10.3389/fcvm.2021.616568

**Published:** 2021-03-30

**Authors:** Xiling Zhang, Xiang Peng, Lulu Li, Huai Yu, Bo Yu

**Affiliations:** ^1^Department of Cardiology, The Second Affiliated Hospital of Harbin Medical University, Harbin, China; ^2^The Key Laboratory of Myocardial Ischemia, Chinese Ministry of Education, Harbin, China

**Keywords:** acute coronary syndrome, statin, smoking, fibrous cap thickness, thin-cap fibroatheroma

## Abstract

**Objective:** This study aimed to investigate the effect of smoking on morphological changes in non-culprit plaques in acute coronary syndrome (ACS) patients at 1 year after percutaneous coronary intervention (PCI), using optical coherence tomography (OCT).

**Background:** Cigarette smoking is an important risk factor for coronary artery disease. However, the reasons for the high risk of re-infarction and worsened health among patients who continue to smoke after PCI remain unclear.

**Methods:** A total of 129 non-culprit plaques were identified from 97 ACS patients who underwent OCT imaging at the time of PCI and at 1-year follow-up. Patients were divided into the following three groups according to their smoking status at 1-year follow-up: persistent smoking group (*n* = 26), smoking cessation group (*n* = 29), and nonsmoking group (*n* = 42). Medical history, serum cholesterol level, coronary angiography data, and OCT-determined plaque morphology were analyzed among the three groups.

**Results:** Relative to baseline levels, the total cholesterol and low-density lipoprotein cholesterol levels significantly decreased in all three groups at 1-year follow-up after statin therapy (*p* < 0.05). The persistent smoking group had a relatively smaller fibrous cap thickness (FCT) and a higher incidence of thin-cap fibroatheroma (TCFA) than the other two groups at 1-year follow-up (*p* < 0.05), although the FCT increased and the incidence of TCFA decreased in all three groups.

**Conclusions:** Persistent smoking is associated with an attenuated effect of statin therapy on plaque stabilization in ACS patients.

## Introduction

Cigarette smoking is one of the most important risk factors for coronary artery disease and can induce coronary artery spasm, coronary artery plaque formation, plaque rupture or erosion, and coronary thrombosis through various mechanisms that lead to acute coronary syndrome (ACS) (1). A report on global and regional cardiovascular mortality indicated that more than 10% of cardiovascular deaths were believed to be due to cigarette smoking (2). An American cohort study reported a higher mortality rate in current smokers with atherosclerosis and ischemic heart disease than in nonsmokers (3). Furthermore, patients who continue to smoke after percutaneous coronary intervention (PCI) have worse outcomes than those who successfully quit (4, 5). Although statins could reduce cardiovascular morbidity and mortality in current smokers, these remain high with respect to absolute incidence compared with those in smoking quitters and never smokers (6).

Owing to its extremely high resolution, optical coherence tomography (OCT) has been widely applied to explore the pathogenesis of ACS. With a resolution 10 times higher than that of intravascular ultrasound, OCT is considered superior for detecting fibrous cap thickness (FCT) and thin-cap fibroatheroma (TCFA) (7). The reasons that patients who continue to smoke after PCI have higher risks of re-infarction and worsened health remain unclear. This study aimed to investigate the effect of smoking on morphological changes in non-culprit plaques in ACS patients at 1 year after PCI using OCT.

## Materials and Methods

### Study Population

We screened 247 patients who underwent OCT from 2009 to 2013 at the Second Affiliated Hospital of Harbin Medical University. Patients who (i) were diagnosed with ACS including ST-elevation myocardial infarction (STEMI), non-STEMI, and unstable angina, (ii) had OCT images of three vessels at the time of PCI and 1-year follow-up, and (iii) had at least one non-culprit/non-target lesion with diameter stenosis >20%, as measured by quantitative coronary angiography (QCA), were included in the study. Patients who (i) had incomplete clinical histories or laboratory data, (ii) had OCT images of poor quality, (iii) underwent coronary artery bypass grafting, (iv) had chronic total occlusion or lesion at the site of previous stenting, and (v) stopped smoking prior to PCI were excluded. Among 247 patients, 97 with 129 non-culprit plaques were included in the analysis.

Patients were divided into the following three groups according to smoking status at the 1-year follow-up: persistent smoking (PS) group (*n* = 26 patients with 32 plaques), smoking cessation (SC) group (*n* = 29 patients with 40 plaques), and nonsmoking (NS) group (*n* = 42 patients with 57 plaques). The definitions of a smoker and a nonsmoker were based on the World Health Organization standard (8). A smoker was defined as an individual who smoked any tobacco product either daily or occasionally at the time of the survey, whereas a nonsmoker referred to an individual who did not smoke at all at the time of the survey. In our study, patients in the PS group were considered smokers who continued to smoke after PCI, whereas patients in the SC group were regarded as smokers who successfully quit smoking after PCI.

### Coronary Angiography Image Acquisition and Analysis

Patients were pretreated with sufficient dual antiplatelet therapy (aspirin and clopidogrel) prior to coronary angiography. Coronary angiography data were analyzed by two investigators blinded to clinical information using the QCA analysis software package (Quantcor QCA version 5.0; Pie Medical Imaging BV, Maastricht, Netherlands). All non-culprit lesions with 20–70% diameter stenosis were included in our analyses. Reference diameter, minimum lumen diameter, diameter stenosis, and lesion length of non-culprit lesions were measured for the analysis.

### OCT Image Acquisition and Analysis

OCT images were obtained using either a time-domain (M2/M3 Cardiology Imaging System; LightLab Imaging, Westford, MA, USA) or frequency-domain OCT system (C7-XR OCT Intravascular Imaging System; St. Jude Medical, St. Paul, MN, USA). Two independent reviewers blinded to clinical information analyzed the OCT images. In the event of disagreement between the two reviewers, a third professional investigator intervened to reach a consensus.

Anatomic marks such as side branches, calcifications, and stent edges were used to identify corresponding lesions on OCT images between baseline and follow-up.

The OCT images were assessed in accordance with the criteria of the Clinical Expert Consensus Document on OCT (9, 10). Lipid was identified as a diffusely bordered signal-poor region with attenuation by the overlying signal-rich layer. Lipid-rich plaque was semiquantified and was defined as a plaque with a lipid arc > 90° and FCT <120 μm. The FCT of each lipid-rich plaque was calculated as the mean thickness of the thinnest three points, as visually estimated. TCFA was defined as a lipid-rich plaque with a maximum lipid arc > 90° and an FCT ≤ 65 mm. A lipid arc was measured at each 1-mm interval throughout the lipid-rich plaque. Moreover, the mean and maximum lipid arcs were recorded. The lipid index was computed by multiplying the mean lipid core arc by the lipid core length. Lipid core length, defined as the length of plaque with > 90° lipid arc, was measured in the longitudinal view. At follow-up, the OCT indexes described above were measured at the same sites via the same methodology according to the landmarks established at baseline.

### Statistical Methods

All statistical analyses were performed using SPSS version 19.0 (IBM Corp., Armonk, NY, USA), and *p-*value < 0.05 was considered statistically significant. For the description of subjects' demographic and clinical characteristics, categorical variables were presented as counts (percentages) and analyzed using chi-squared test or Fisher's exact test. Continuous variables were expressed as mean ± standard deviation. Differences among the three groups were analyzed using analysis of variance or Kruskal–Wallis H test. Multiple comparisons were followed by *post hoc* tests and Bonferroni correction if the differences among the three groups were statistically significant, and *p* < 0.017 was considered statistically significant. Owing to the presence of multiple plaques within a single subject, a generalized estimating equation approach was employed to identify independent predictors for the presence of TCFA and FCT. Variables included in the model were age, gender, hypertension, hyperlipidemia, diabetes mellitus (DM), statin use, and low-density lipoprotein cholesterol (LDL-C) level.

## Results

### Study Population

**Figure 1:**
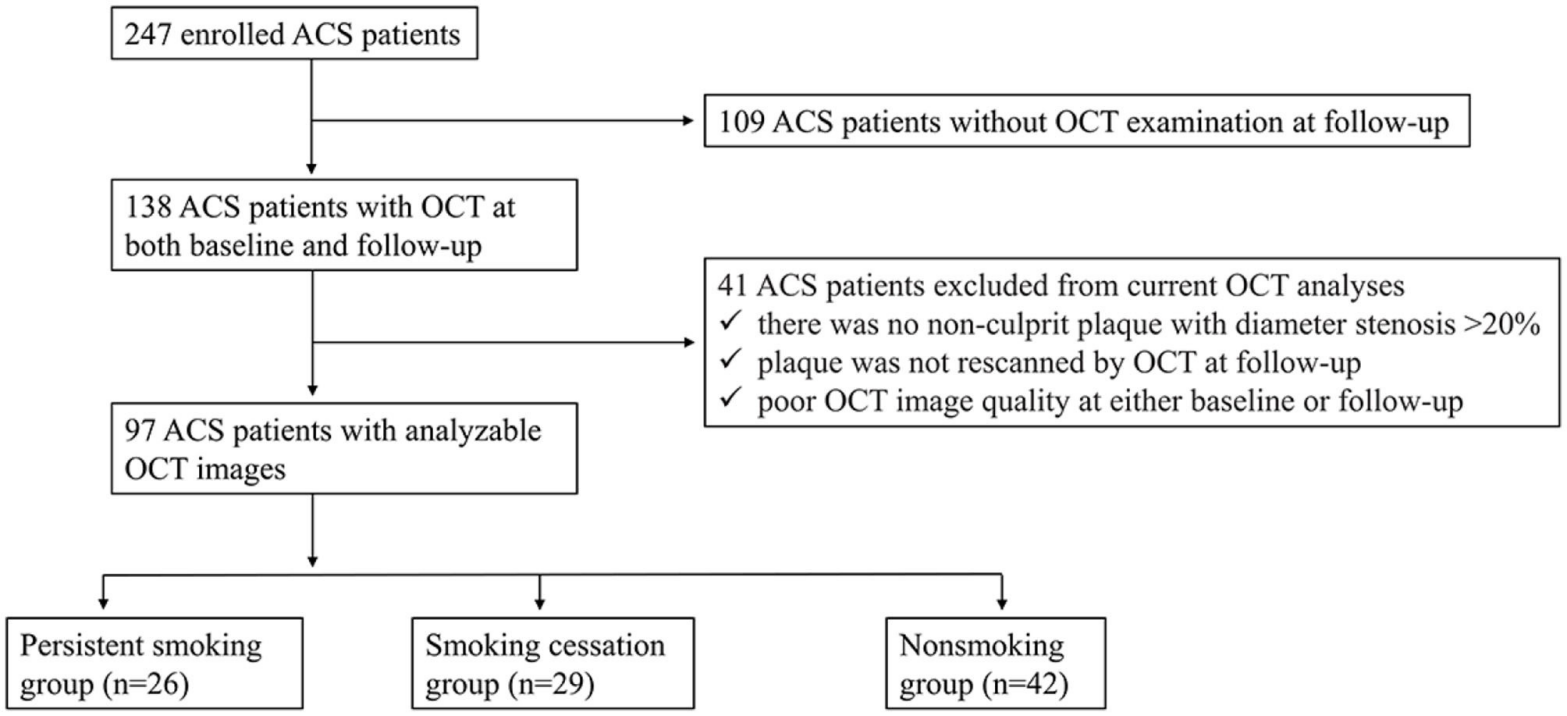
Flow chart showing patient enrollment. ACS, acute coronary syndrome; OCT, optical coherence tomography.

A flow chart categorizing ACS patients based on their smoking status is presented in Figure 1. The baseline characteristics of study patients are summarized in Table 1. Significant differences in age (52.9 ± 6.5 years vs. 53.0 ± 11.9 years vs. 58.2 ± 7.5 years, *p* = 0.011) and gender (81 vs. 79 vs. 38%, *p* < 0.001) were observed among the three groups. Although the difference in age was not significant after multiple comparisons, smokers (including patients in both PS and SC groups) seemed to be younger than nonsmokers. Smokers comprised a higher proportion of males than nonsmokers (*p* = 0.001). Smokers with DM were fewer than nonsmokers (19 vs. 45% vs. 62%, *p* = 0.002). There was no difference in body mass index or hypertension among the three groups. More than 90% of patients were administered with aspirin, clopidogrel, and statin after PCI; nonetheless, the proportion of patients treated with statin did not significantly differ among the three groups. There was no change in antiplatelet and statin therapy at the 1-year follow-up among the three groups.

**Table 1:** Baseline characteristics.

**Variable**	**PS group (*n* = 26)**	**SC group (*n* = 29)**	**NS group (*n* = 42)**	***p*-value**	***p-*****value**		
					**PS vs. SC**	**PS vs. NS**	**SC vs. NS**
Age, years	52.9 ± 6.5	53.0 ± 11.9	58.2 ± 7.5	0.011	1.000	0.038	0.032
Men, *n* (%)	21 (80.7%)	23 (79.3%)	16 (38.1%)	<0.001	0.893	0.001	0.001
BMI, kg/m^2^	24.8 ± 2.9	25.2 ± 3.1	25.0 ± 2.6	0.838			
STEMI, *n* (%)	2 (7.7%)	9 (31.0%)	4 (9.5%)	0.033	0.031	0.796	0.021
Hypertension, *n* (%)	14 (53.8%)	18 (62.0%)	27 (64.3%)	0.683			
DM, *n* (%)	5 (19.2%)	13 (44.8%)	26 (61.9%)	0.002	0.043	0.001	0.155
Statin, *n* (%)	24 (92.3%)	29 (100.0%)	40 (95.2%)	0.287			
Rosuvastatin 10 mg	5 (19.2%)	3 (10.3%)	13 (30.9%)	0.110			
Atorvastatin 20 mg	9 (34.6%)	14 (48.3%)	16 (38.1%)	0.548			
Atorvastatin 60 mg	5 (19.2%)	12 (41.4%)	9 (21.4%)	0.104			
Simvastatin 20 mg	5 (19.2%)	0 (0.0%)	1 (2.4%)	0.005			
Aspirin, *n* (%)	26 (100.0%)	29 (100.0%)	42 (100.0%)	1.000			
Clopidogrel, *n* (%)	25 (96.15%)	29 (100.00%)	42 (100.00%)	0.268			
ACEI/ARB, *n* (%)	12 (46.2%)	16 (55.2%)	18 (42.8%)	0.587			
Beta-blocker, *n* (%)	16 (61.5%)	16 (55.2%)	28 (66.7%)	0.618			

*Data are presented as n (%) or mean ± SD. PS, persistent smoking; SC, smoking cessation; NS, non-smoking; BMI, body mass index; STEMI, ST-elevation myocardial infarction; DM, diabetes mellitus; ACEI, angiotensin-converting enzyme inhibitor; ARB, angiotensin II receptor blocker*.

### Laboratory Findings

**Figure 2:**
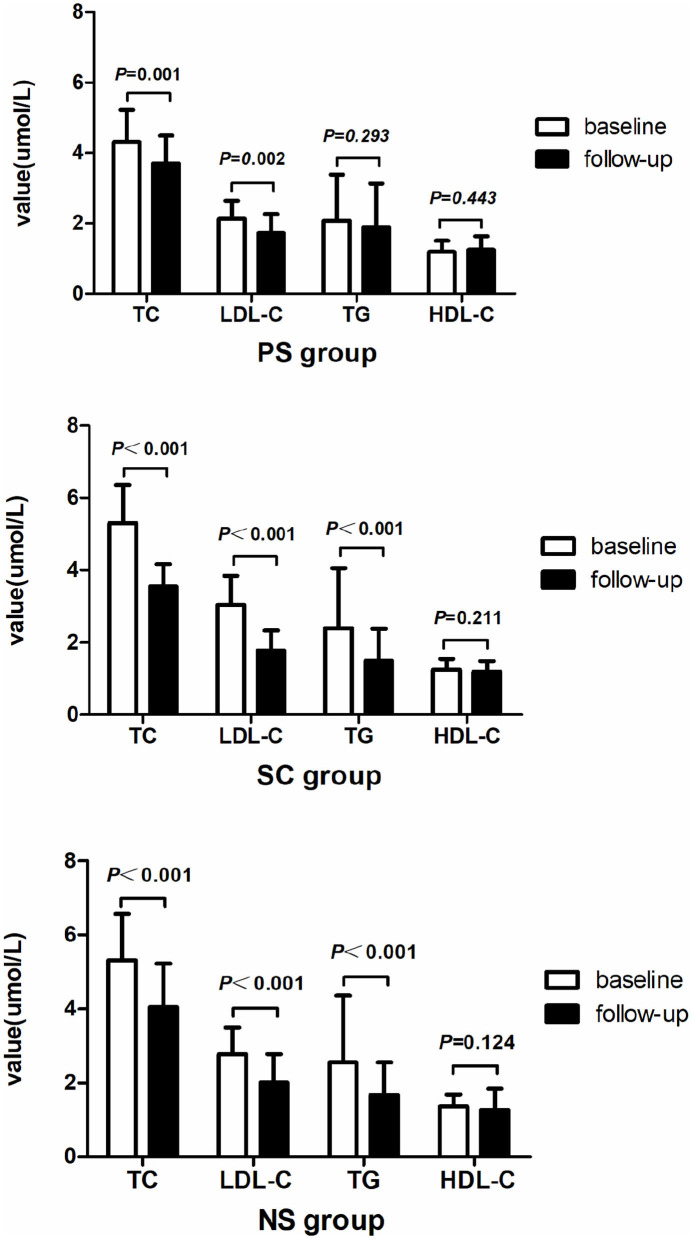
Changes in cholesterol level in each group observed after 1 year. TC and LDL-C levels decreased in all three groups after 1 year of statin therapy. TG level significantly decreased in the SC and NS groups, but not in the PS group. TC, total cholesterol; LDL-C, low-density lipoprotein cholesterol; TG, triglyceride; HDL-C, high-density lipoprotein cholesterol.

The laboratory findings are presented in Table 2 and Figure 2. The total cholesterol (TC) and LDL-C levels were significantly different among the three groups at baseline (TC: 4.32 ± 0.9 mmol/L vs. 5.31 ± 1.05 mmol/L vs. 5.31 ± 1.26 mmol/L, *p* < 0.001; LDL-C: 2.13 ± 0.51 mmol/L vs. 3.04 ± 0.81 mmol/L vs. 2.78 ± 0.71 mmol/L, *p* < 0.001). The TC and LDL-C levels were lower in the PS group than in the SC and NS groups (*p* < 0.017). However, differences in the TC and LDL-C levels at the 1-year follow-up were not significant. Relative to the baseline, the TC and LDL-C levels significantly decreased in all three groups at the 1-year follow-up (*p* < 0.05). The change in TC level was smaller in the PS group than in the SC group (0.63 ± 1.05 mmol/L vs. 1.75 ± 1.11 mmol/L, *p* = 0.001). The triglyceride (TG) level did not significantly change in the PS group but significantly decreased in the SC and NS groups at the 1-year follow-up (SC: 2.40 ± 1.66 mmol/L vs. 1.51 ± 0.87 mmol/L, *p* < 0.001; NS: 2.56 ± 1.80 mmol/L vs. 1.68 ± 0.88 mmol/L, *p* < 0.001).

**Table 2:** Laboratory findings for the three groups at baseline and 1-year follow-up.

**Variable**	**Time**	**PS group (*n* = 26)**	**SC group (*n* = 29)**	**NS group (*n* = 42)**	***p*-value**	***p-*****value**		
						**PS vs. SC**	**PS vs. SC**	**PS vs. SC**
TC, mmol/L	Baseline	4.32 ± 0.9	5.31 ± 1.05	5.31 ± 1.26	<0.001	0.004	0.002	1.000
	Follow-up	3.7 ± 0.79	3.56 ± 0.62	4.05 ± 1.17	0.084			
	Change over time	0.63 ± 1.05	1.75 ± 1.11	1.27 ± 1.12	0.001	0.001	0.063	0.211
LDL-C, mmol/L	Baseline	2.13 ± 0.51	3.04 ± 0.81	2.78 ± 0.71	<0.001	<0.001	0.001	0.359
	Follow-up	1.72 ± 0.55	1.79 ± 0.55	2.01 ± 0.77	0.159			
	Change over time	0.41 ± 0.73	1.25 ± 0.91	0.77 ± 0.87	0.002	0.020	0.488	0.302
TG, mmol/L	Baseline	2.08 ± 1.31	2.40 ± 1.66	2.56 ± 1.80	0.508			
	Follow-up	1.90 ± 1.23	1.51 ± 0.87	1.68 ± 0.88	0.333			
	Change over time	0.19 ± 0.98	0.90 ± 1.23	0.88 ± 1.53	0.073			
HDL-C, mmol/L	Baseline	1.19 ± 0.32	1.25 ± 0.30	1.36 ± 0.33	0.082			
	Follow-up	1.25 ± 0.39	1.2 ± 0.3	1.27 ± 0.57	0.810			
	Change over time	−0.05 ± 0.35	0.05 ± 0.34	0.09 ± 0.51	0.374			

*Data are presented as mean ± SD. PS, persistent smoking; SC, smoking cessation; NS, non-smoking; TC, total cholesterol; LDL-C, low-density lipoprotein cholesterol; TG, triglyceride; HDL-C, high-density lipoprotein cholesterol*.

### Angiographic Findings

Lesion location and angiographic findings are listed in Table 3. Diameter stenosis was similar among the three groups at baseline but seemed to have increased in the PS group compared with that in the other groups (*p* = 0.05). No significant difference in lesion location was observed among the three groups. Furthermore, no significant differences in minimum lumen diameter, reference diameter, and lesion length were noted among the three groups at either baseline or 1-year follow-up.

**Table 3:** QCA data.

**QCA variable**	**Time**	**PS group (*n* = 32)**	**SC group (*n* = 40)**	**NS group (*n* = 57)**	***p*-value**
Coronary plaque location					0.269
Right		12 (37.5%)	14 (35%)	30 (52.63%)	
Left anterior descending		14 (43.75%)	15 (37.5%)	14 (24.56%)	
Left circumflex		6 (18.75%)	11 (27.5%)	13 (22.81%)	
Minimum lumen diameter, mm	Baseline	1.96 ± 0.59	1.97 ± 0.60	2.04 ± 0.43	0.750
	Follow-up	2.03 ± 0.60	2.05 ± 0.62	2.04 ± 0.47	0.982
Reference diameter, mm	Baseline	2.83 ± 0.60	2.71 ± 0.57	2.85 ± 0.54	0.465
	Follow-up	2.84 ± 0.59	2.75 ± 0.67	2.79 ± 0.59	0.817
Lesion length, mm	Baseline	11.06 ± 2.90	10.41 ± 3.17	10.23 ± 2.78	0.433
	Follow-up	11.06 ± 2.69	10.60 ± 3.30	10.38 ± 3.49	0.641
Diameter stenosis, %	Baseline	31.09 ± 10.68	28.13 ± 12.67	28.39 ± 9.20	0.438
	Follow-up	32.58 ± 13.82	25.48 ± 11.95	27.86 ± 11.44	0.050

*Data are presented as n (%) or mean ± SD. QCA, quantitative coronary angiography; PS, persistent smoking; SC, smoking cessation; NS, non-smoking*.

### OCT Findings

**Figure 3:**
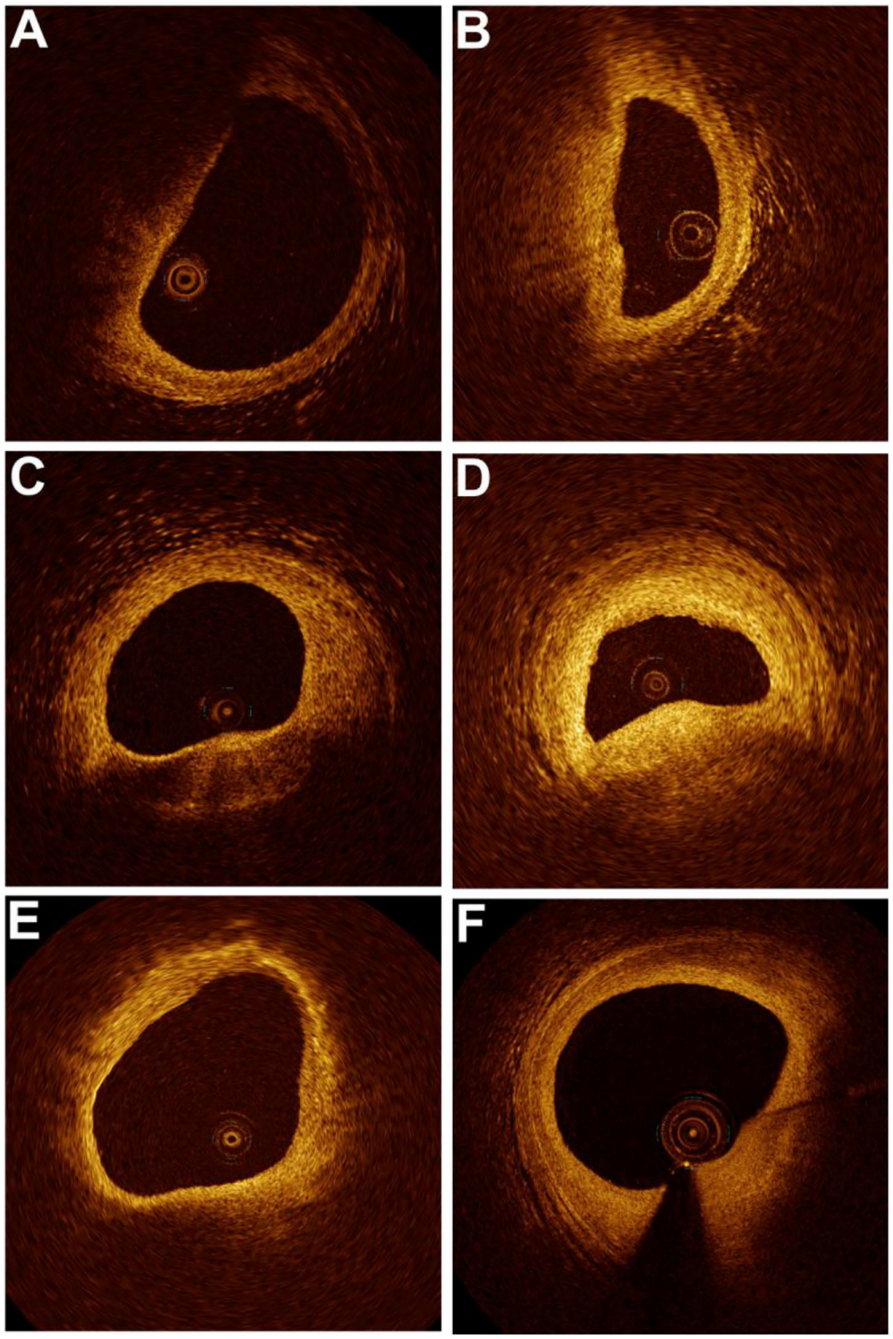
Changes in plaque characteristics for each group at baseline and 1-year follow-up. In the persistent smoking group, the fibrous cap thickness (FCT) was 0.05 mm with a lipid arc of 117.9° at baseline **(A)** and 0.08 mm with a lipid arc of 161.5° at the 1-year follow-up **(B)**. In the smoking cessation group, the FCT was 0.04 mm with a lipid arc of 138.3° at baseline **(C)** and 0.11 mm with a lipid arc of 95.7° at the 1-year follow-up **(D)**. In the nonsmoking group, the FCT was 0.11 mm with a lipid arc of 235.7° at baseline **(E)** and 0.25 mm with a lipid arc of 157.3° at the 1-year follow-up **(F)**.

**Figure 4:**
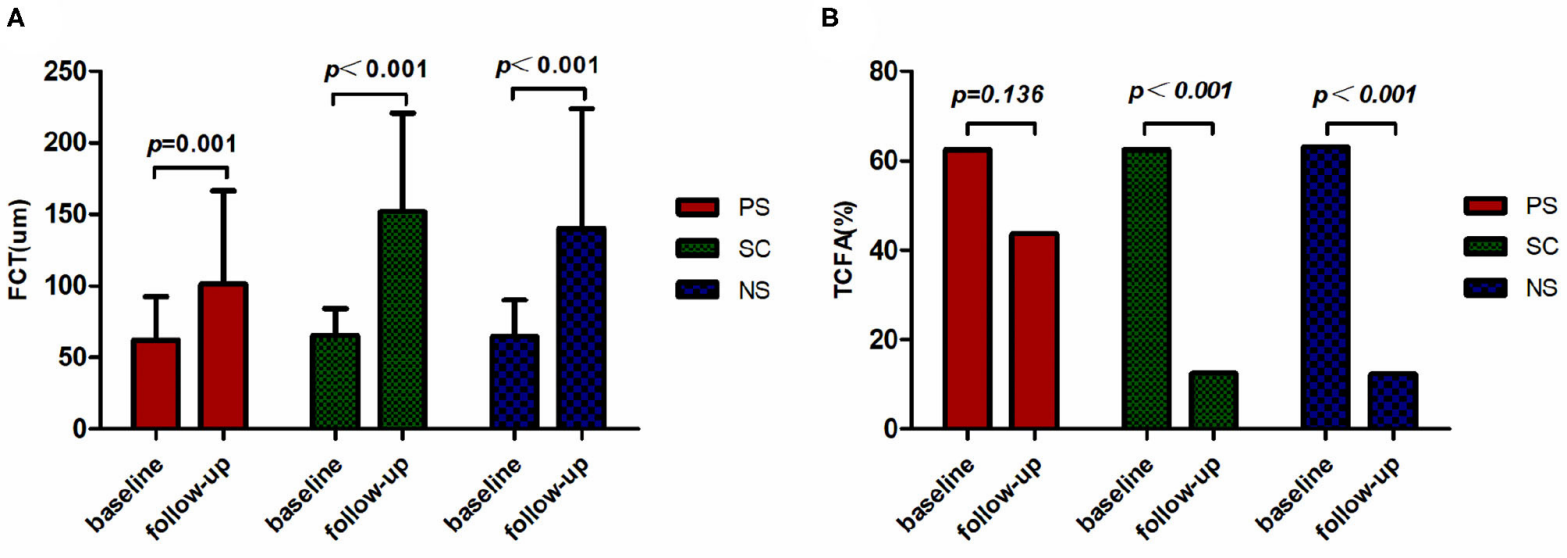
Changes in FCT and TCFA. The FCT **(A)** and incidence of TCFA **(B)** assessed by OCT at two time points are presented for each group. The FCT in all three groups was larger at the 1-year follow-up. The incidence of TCFA at 1 year declined relative to the baseline in the SC and NS groups. FCT, fibrous cap thickness; TCFA, thin-cap fibroatheroma; PS, persistent smoking; SC, smoking cessation; NS, nonsmoking.

The plaque characteristics identified by OCT are presented in Table 4 and Figure 3. Differences in FCT and TCFA between baseline and 1-year follow-up for each of the three groups are shown in Figure 4. The plaque amount per patient was similar among the three groups. Furthermore, no significant differences in FCT, lipid index, maximum lipid arc, lipid core length, and TCFA were observed among the three groups at baseline. FCT significantly increased in all three groups at the 1-year follow-up (PS: 62.44 ± 30.40 μm vs. 101.47 ± 65.13 μm, *p* = 0.001; SC: 65.60 ± 18.96 μm vs. 152.18 ± 68.69 μm, *p* < 0.001; NS: 65.14 ± 25.15 μm vs. 140.37 ± 83.46 μm, *p* < 0.001), although the change in FCT was relatively smaller in the PS group than in the other groups at the 1-year follow-up (*p* = 0.016). The incidence of TCFA decreased in the SC and NS groups at the 1-year follow-up (SC: 62.50 vs. 12.5%, *p* < 0.001; NS: 63.16% vs. 12.28%, *p* < 0.001); however, the incidence of TCFA remained higher in the PS group at the 1-year follow-up (*p* = 0.006). The lipid index and lipid core length were higher in the PS group than in the SC group at the 1-year follow-up (*p* = 0.011). Compared with the lipid index at baseline, lipid index at the 1-year follow-up decreased in all three groups (*p* < 0.05). There was no significant difference in the changes in mean lipid arc and maximum lipid arc among the three groups.

**Table 4:** OCT findings.

**OCT**	**Time**	**PS group (*n* = 32)**	**SC group (*n* = 40)**	**NS group (*n* = 57)**	***p*-value**	***p-*****value**		
						**PS vs. SC**	**PS vs. SC**	**PS vs. SC**
Average plaque number		1.23 ± 0.514	1.38 ± 0.862	1.36 ± 0.759	0.721			
FCT, μm	Baseline	62.44 ± 30.40	65.60 ± 18.96	65.14 ± 25.15	0.878			
	Follow-up	101.47 ± 65.13	152.18 ± 68.69	140.37 ± 83.46	0.016	0.015	0.060	0.830
TCFA, *n* (%)	Baseline	20 (62.50%)	25 (62.50%)	36 (63.16%)	0.988			
	Follow-up	14 (43.75%)	5 (12.5%)	7 (12.28%)	0.006	0.003	0.001	0.605
Lipid index	Baseline	2648.83 ± 1962.41	1780.44 ± 1509.66	1970.88 ± 1709.79	0.088			
	Follow-up	2215.43 ± 1654.17	1330.92 ± 1030.50	1557.96 ± 1135.04	0.011	0.011	0.058	1.000
Lipid core length, mm	Baseline	12.77 ± 7.53	10.18 ± 6.32	10.28 ± 6.32	0.339			
	Follow-up	11.76 ± 7.67	8.50 ± 3.9 4	8.82 ± 5.16	0.028	0.045	0.056	1.000
Mean lipid arc, °	Baseline	190.78 ± 57.61	167.49 ± 57.76	180.46 ± 54.41	0.380			
	Follow-up	178.61 ± 51.49	147.99 ± 68.13	168.85 ± 63.19	0.140			
Maximum lipid arc, °	Baseline	264.48 ± 79.78	224.49 ± 74.92	242.12 ± 74.56	0.206			
	Follow-up	239.72 ± 71.84	193.82 ± 86.57	222.16 ± 86.37	0.071			

*Data are presented as n (%) or mean ± SD. PS, persistent smoking; SC, smoking cessation; NS, non-smoking; FCT, fibrous cap thickness; TCFA, thin-cap fibroatheroma*.

### Generalized Estimating Equation Results

After adjustment for age, gender, hypertension, DM, statin use, and LDL-C level, TCFA was more frequently observed in the PS group than in the SC and NS groups (PS vs. SC, *p* = 0.034; PS vs. NS, *p* = 0.011). FCT was lower in the PS group than in the NS group (PS vs. NS, *p* = 0.005), whereas FCT did not differ between the PS and SC groups (PS vs. SC, *p* = 0.295).

## Discussion

The main findings of the present study were as follows: (i) statin therapy could increase FCT and decrease the incidence of TCFA and the lipid index of non-culprit plaques in ACS patients regardless of smoking; (ii) patients who continued to smoke after PCI had a relatively smaller FCT and a higher incidence of TCFA than patients who stopped smoking or never smoked at the 1-year follow-up; and (iii) the changes in FCT, TCFA, and lipid index were similar between the SC and NS groups. After adjustment for age, gender, hypertension, DM, statin use, and LDL level, smoking was independently correlated with a higher incidence of TCFA and a relatively smaller FCT after PCI.

### Effect of Statin on LDL-C and Plaque

Pathological studies have confirmed that LDL-C, particularly oxidized LDL, could induce atherosclerosis by stimulating monocyte infiltration as well as smooth muscle cell migration and proliferation (11). A previous study reported the association between the severity of LDL-C level elevation and a higher risk of mortality among healthy subjects (12). The relative risk of major cardiovascular events decreases by 23% for every 1 mmol/L reduction in LDL-C level (13). Statin therapy can reduce cardiovascular morbidity and mortality regardless of gender and risk (14, 15) and all-cause mortality in patients with lower LDL-C level (16). Statins have been proven to reduce the frequency of TCFA and plaque volume by decreasing serum atherogenic lipoproteins and inflammatory biomarkers (17–19). In our study, the TC and LDL-C levels decreased in all three groups after 1 year of statin therapy. Moreover, the FCT increased, whereas the incidence of TCFA decreased, which were consistent with the results of previous studies mentioned above.

### Cigarette Smoking Affects Lipid and Lipoprotein Levels

Campbell et al. (20) summarized the unfavorable effects of cigarette smoking on lipid and lipoprotein levels and reported that cigarette smoking could increase the TC, TG, and LDL-C levels. Furthermore, cigarette smoking can alter the critical enzymes for lipid transport, lowering lecithin–cholesterol acyltransferase activity and altering both cholesteryl ester transfer protein and hepatic lipase activities (21). These factors can favor the progression of atherosclerosis. Although the TC, LDL-C, and TG levels declined in all three groups at the 1-year follow-up, the changes in TC, LDL-C, and TG levels were less considerable in the PS group than in the SC and NS groups. Nevertheless, the PS group had a relatively smaller FCT and a higher incidence of TCFA than the other groups. Based on the conclusion of previous studies, the findings of our study indicate that cigarette smoking attenuated the stabilizing effect of statin on the vulnerable plaque.

### Effect of Cigarette Smoking on Plaque Vulnerability

Plaque vulnerability is a major risk factor for ACS and depends on the plaque's lipid composition, FCT, degradation of extracellular matrix proteins, inflammatory cell recruitment, and intraplaque hemorrhage (22). Cigarette smoking has been shown to be independently associated with lipid-rich plaques and contributes to an increased risk of plaque vulnerability (23, 24). As the major component of cigarette, nicotine has been indicated to potentiate the proatherogenic effects of oxidized low-density lipoprotein (oxLDL) by stimulating and upregulating macrophage CD36 signaling (25). Thus, cigarette smoking potentiates endothelial dysfunction by enhancing oxLDL (26). Cigarette smoking can downregulate n-prolyl-4-hydroxylase, which contributes to the development of a thin fibrous cap in atherosclerotic plaques among smokers (27). Biglycan has recently been indicated as a major source of low-density lipoprotein in the normal arterial intima–media layer. Biglycan mRNA can be enhanced by cigarette smoking and appears to be associated with a proatherogenic profile (28). Cigarette smoking induces inflammation and oxidative stress, potentially increasing matrix metalloproteinase expression within atherosclerotic plaques, particularly at the shoulder regions, in which increased stress and matrix degradation can result in fibrous cap rupture (29). In our study, the PS group had more TCFA, relatively smaller FCT, and higher lipid index at 1-year follow-up than the SC and NS groups. TCFA has been considered a strong predictor of ACS (30). The results of our study might explain why smoking patients are at a higher risk of major adverse cardiovascular events after PCI.

### Smoking Status Affects the Prognosis of Patients After PCI

Approximately one-third of ACS patients who smoke cannot quit smoking even after PCI, which worsens their outcomes compared with those of patients who successfully quit. An analysis from the Acute Catheterization and Urgent Intervention Triage Strategy trial revealed that smoking might be an independent predictor of high 1-year mortality among patients presenting with non-ST-elevation ACS (31). Other studies reported that patients who continued to smoke after PCI were at higher risk of Q-wave infarction and death than nonsmokers and that smoking cessation either before or after PCI was beneficial (4, 32). Smoking cessation could persistently improve endothelial function, which may exert a mediating effect and reduce the risk of cardiovascular diseases (33). Cigarette smoking has a broad impact on genome-wide methylation, by which cigarette smoking predisposes smokers to heart disease. Nonetheless, former smokers have fewer methylated genes than current smokers, indicating persistent altered methylation with attenuation after cessation (34). In our study, the TC, LDL-C, and TG levels notably declined in the SC group, and the SC group had a larger FCT and a lower incidence of TCFA than persistent smokers. These results were similar to those of the NS group, suggesting that SC combined with statin therapy could stabilize the vulnerable plaque.

### Study Limitations

This study has several limitations. First, it is a retrospective, single-center study with a small sample; therefore, it is limited by the possibility of selection bias. Our study necessitated subjects to have both baseline and 1-year OCT images, and the OCT images were also required to include non-culprit coronary arteries; all of these limited the sample size. Second, passive smoking history, as well as the quantification and duration of smoking history, was not considered in this study. Third, the ostial and distal segments of the coronary arteries were not considered in our study because of OCT imaging limitations, which might lead to bias. Finally, owing to the limitations of the current OCT imaging system, some fibrous plaques might have been misdiagnosed as TCFA.

## Conclusions

In the present study, although statin therapy decreased the TC and LDL-L levels and stabilized the plaque, patients who continued smoking after PCI had a relatively smaller FCT and a higher incidence of TCFA than patients in the SC and NS groups. These results indicate that cigarette smoking might attenuate the stabilizing effect of statin on the vulnerable plaque and might explain why smokers have worse endpoints than nonsmokers and smoking quitters. The results highlight the importance of providing early SC aids to patients who smoke.

## Data Availability Statement

The raw data supporting the conclusions of this article will be made available by the authors, without undue reservation.

## Ethics Statement

The studies involving human participants were reviewed and approved by the ethics committee of the Second Affiliated Hospital of Harbin Medical University (Harbin, China). Written informed consent for participation was not required for this study in accordance with the national legislation and the institutional requirements.

## Author Contributions

XZ and XP made substantial contributions to conception, design, and planning of the study. LL and HY participated in interpretation of data and statistical analyses. BY were responsible for the overall content and served as guarantor. All authors were involved in reporting the results of this study and read and approved the final version of the submitted manuscript.

## Conflict of Interest

The authors declare that the research was conducted in the absence of any commercial or financial relationships that could be construed as a potential conflict of interest.
